# Supplementary material for: Contribution of Uremia to Ureaplasma-Induced Hyperammonemia
Source: Microbiol Spectr. 2022 Feb 16;10(1):e01942-21. doi: 10.1128/spectrum.01942-21 (PMC8849080; doi:10.1128/spectrum.01942-21)
Supplement: SUPPLEMENTAL FILE 1 — Supplemental material. Download SPECTRUM01942-21_Supp_1_seq9.pdf, PDF file, 0.1 MB [file spectrum01942-21_supp_1_seq9.pdf]

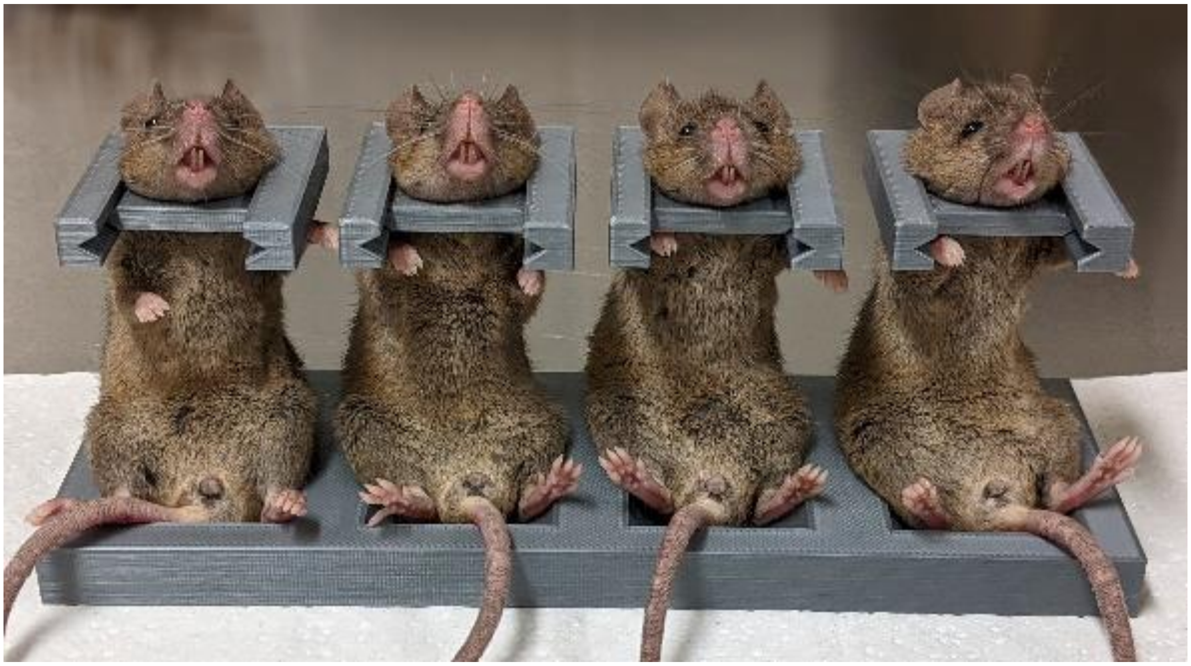

Supplemental Figure 1. Murine vertical stabilization apparatus. STL file (for 3D printing) available upon request.

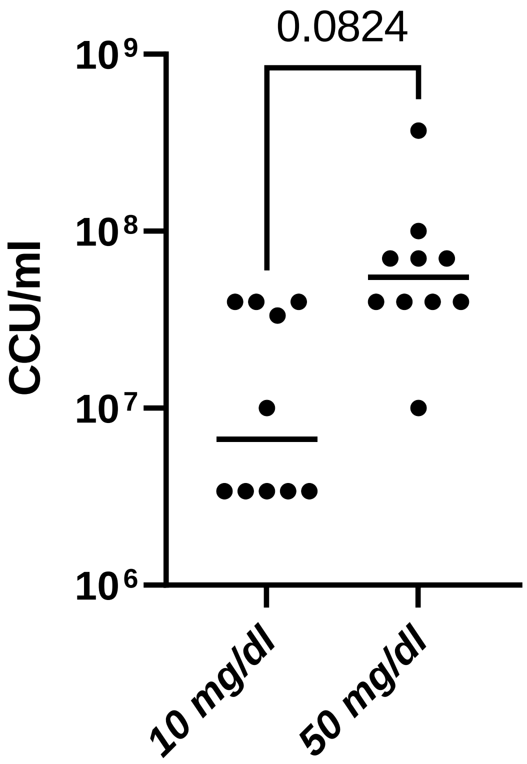

Supplemental Figure 2. Endpoint *Ureaplasma* quantities between normal and uremic conditions. Isolates of *Ureaplasma parvum* or *urealyticum* (N=3 per isolate per condition) were grown in the dialyzed flow system under normal (10 mg/dL urea) or uremic (50 mg/dL urea) conditions for 24 hours. Endpoint color changing units (CCUs) were quantified in triplicate via serial dilution in 10B broth (Remel) containing phenol red. The average positive color change from yellow to fushcia at the highest dilution factor was calculated. Significance (p-value) between conditions was determined via a two-tailed unpaired t-test.

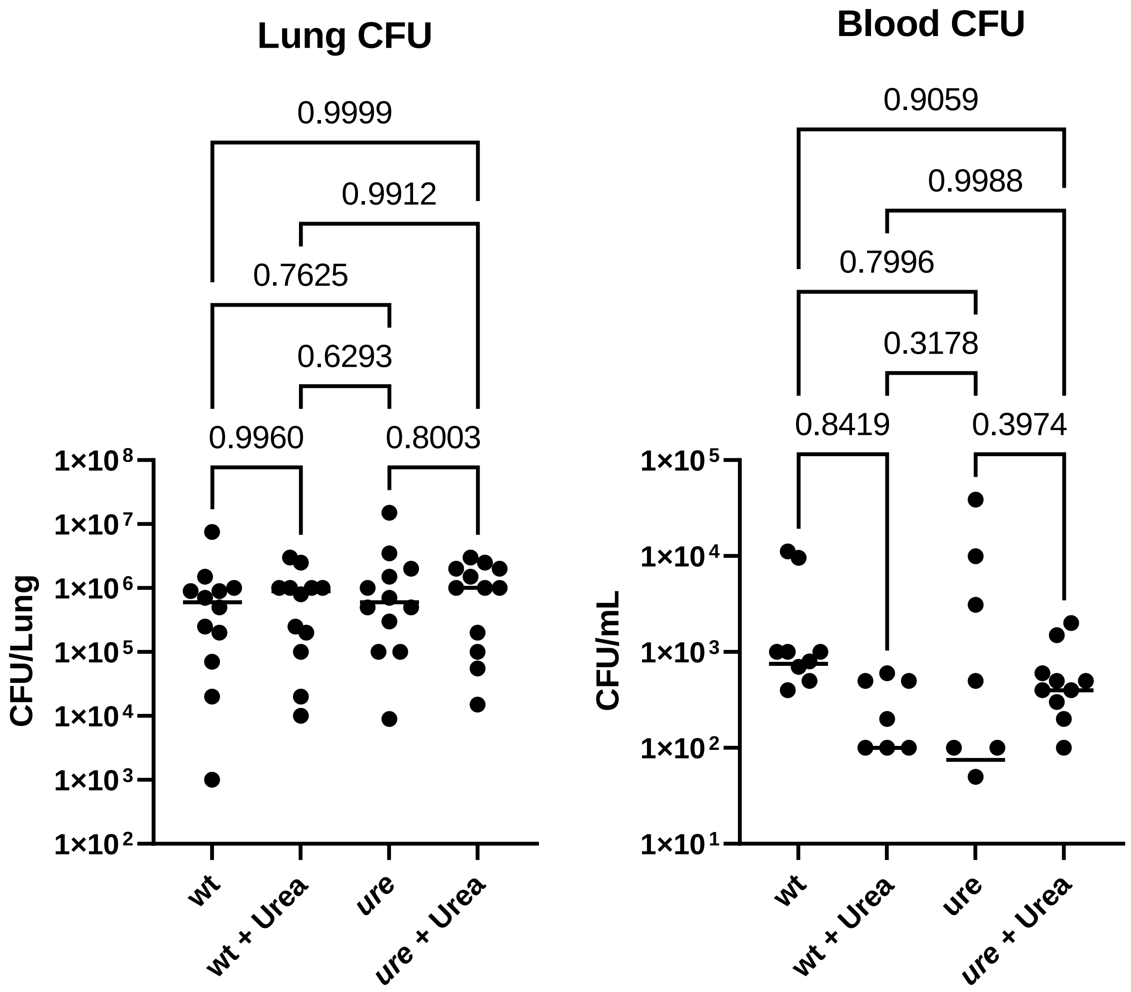

Supplemental Figure 3. Endpoint *Escherichia coli* lung and blood quantities. Urea-fed (+ Urea) and normal mice were infected intraperitoneally and intratracheally with either wild-type (wt) or urease-positive *E. coli* (*ure*). CFUs were measured by standard 1:10 serial dilution and 20 µL plating on tryptic soy agar. Significance (p-values) was determined via one-way ANOVA.
